# Lower plasma trans-4-hydroxyproline and methionine sulfoxide levels are associated with insulin dysregulation in horses

**DOI:** 10.1186/s12917-018-1479-z

**Published:** 2018-05-02

**Authors:** Ákos Kenéz, Tobias Warnken, Karsten Feige, Korinna Huber

**Affiliations:** 1 Institute of Animal Science, Faculty of Agricultural Sciences, University of Hohenheim, Fruwirthstraße 35, 70593 Stuttgart, Germany; 2 Clinic for Horses, University of Veterinary Medicine Hannover, Foundation, Bünteweg 9, 30559 Hannover, Germany; 3 Present address: College of Veterinary Medicine and Life Sciences, City University of Hong Kong, Kowloon Tong, Hong Kong SAR

**Keywords:** Metabolome, Metabolomics, Insulin sensitivity, Insulin dysregulation, Oral glucose test, Horses

## Abstract

**Background:**

Insulin dysregulation in horses is a metabolic condition defined by high insulin concentrations in the blood and peripheral insulin resistance. This hyperinsulinemia is often associated with severe damage in the hooves, resulting in laminitis. However, we currently lack detailed information regarding the potential involvement of particular metabolic pathways in pathophysiological causes and consequences of equine insulin dysregulation. This study aimed to assess the dynamic metabolic responses given to an oral glucose test (OGT) in insulin-sensitive and insulin-dysregulated horses by a targeted metabolomics approach to identify novel metabolites associated with insulin dysregulation.

**Results:**

Oral glucose testing triggered alterations in serum insulin (26.28 ± 4.20 vs. 422.84 ± 88.86 μIU/mL, *p* < 0.001) and plasma glucose concentrations (5.00 ± 0.08 vs. 9.43 ± 0.44 mmol/L, *p* < 0.001) comparing basal and stimulated conditions after 180 min. Metabolome analyses indicated OGT-induced changes in short-chain acylcarnitines (6.00 ± 0.53 vs. 3.99 ± 0.23 μmol/L, *p* < 0.001), long-chain acylcarnitines (0.13 ± 0.004 vs. 0.11 ± 0.002 μmol/L, *p* < 0.001) and amino acids (2.18 ± 0.11 vs. 1.87 ± 0.08 μmol/L, *p* < 0.05). Kynurenine concentrations increased (2.88 ± 0.18 vs. 3.50 ± 0.19 μmol/L, *p* < 0.01), whereas spermidine concentrations decreased during OGT (0.09 ± 0.004 vs. 0.08 ± 0.002 μmol/L, *p* < 0.01), indicating proinflammatory conditions after oral glucose load. Insulin dysregulation was associated with lower concentrations of trans-4-hydroxyproline (4.41 ± 0.29 vs. 6.37 ± 0.71 μmol/L, *p* < 0.05) and methionine sulfoxide (0.40 ± 0.06 vs. 0.87 ± 0.13 μmol/L, *p* < 0.01; mean ± SEM in insulin-dysregulated vs. insulin-sensitive basal samples, respectively), two metabolites which are related to antioxidant defense mechanisms.

**Conclusion:**

Oral glucose application during OGT resulted in profound metabolic and proinflammatory changes in horses. Furthermore, insulin dysregulation was predicted in basal samples (without OGT) by pathways associated with trans-4-hydroxyproline and methionine sulfoxide, suggesting that oxidative stress and oxidant–antioxidant disequilibrium are contributing factors to insulin dysregulation. The present findings provide new hypotheses for future research to better understand the underlying pathophysiology of insulin dysregulation in horses.

**Electronic supplementary material:**

The online version of this article (10.1186/s12917-018-1479-z) contains supplementary material, which is available to authorized users.

## Background

The equine metabolic syndrome (EMS) in horses is associated with severe disturbances in glucose and lipid homeostasis and adiposity. Equine insulin dysregulation reflected by hyperinsulinemia is a key symptom [1, 2]. However, the underlying causal pathomechanisms of EMS and metabolic consequences of insulin dysregulation are currently poorly understood. Laminitis, a chronic painful disease of the hooves, develops as a severe consequence and is often a cause for euthanasia [3]. Insulin and glucose concentrations in plasma as indicators of metabolic dysregulation are frequently measured parameters in the clinical routine of equine medicine. Oral glucose tests and other challenges were used to assess insulin dysregulation exactly [1]; however, these tests assay only the disturbed insulin response, but do not provide information about potential causal pathways leading to insulin dysregulation in horses. Furthermore, data on the metabolic consequences of equine insulin dysregulation, other than hyperinsulinemia, are still limited.

Metabolomics can be used as a powerful tool to depict the phenotypic image of metabolism under the current conditions [4]. This is provided by a snapshot-like quantification of an extensive set of metabolites which are products or substrates of various metabolic pathways. Due to the known links between metabolites and pathways, hypotheses about underlying (patho)physiological processes can be proposed. Furthermore, new hypotheses concerning affected pathways can be established as a basis for new experimental designs by introducing novel associations between certain metabolites and phenotypic observations. These new scientific studies will help to identify pathophysiological causes or consequences of equine insulin dysregulation. The identification of potential biomarkers capturing the acute health status is another benefit of metabolomics approaches.

Therefore, the aims of the present study were to (1) elucidate the dynamic metabolic response to a defined oral glucose challenge in horses previously classified as insulin-sensitive (IS) or -dysregulated (ID), and (2) to identify novel metabolites associated with the ID state. To accomplish these aims, the blood plasma metabolome of IS and ID horses was analyzed using a targeted metabolomics approach, important metabolites were selected by multivariate statistical methods, and pathways associated with the selected metabolites were described based on previous knowledge in the context of insulin homeostasis and metabolic disorders.

## Methods

### Animals and sample collection

Twenty horses of various breeds, ages and body weights (BW) were included in the study. Body condition scoring (BCS) (according to Henneke et al. [5]), (BW) and ages were recorded for each horse. The ages of the horses ranged from 6 to 23 years, and their BW ranged from 147 to 695 kg. Horses had unknown insulinemic status prior to testing and blood samples were collected during routine diagnostic procedures for the assessment of insulin dysregulation in the Clinic for Horses (University of Veterinary Medicine, Hannover). Informed consent was obtained from the owners for scientific use and publication. Horses were fasted overnight before sampling and, in connection with the sample collection, an oral glucose testing (OGT) procedure [6] was conducted. The OGT was carried out by administering 1.0 g/kg BW glucose powder dissolved in 2 L of water by a nasogastric tube. Blood samples were collected from each horse three times: immediately before starting the OGT (BASAL), 120 min after starting the OGT (OGT-120) and 180 min after starting the OGT (OGT-180). Samples were collected via a jugular vein catheter, transferred into plain tubes (Vacuette® Greiner Bio One, Frickenhausen, Germany) for serum and EDTA tubes (Vacuette® Greiner Bio One, Frickenhausen, Germany) for plasma preparation. Blood for serum was incubated at room temperature for 1 h before centrifugation at 3000 g for 6 min at room temperature and blood for plasma was immediately centrifuged at 3000 g for 6 min. Serum and plasma samples were shock-frozen in liquid nitrogen and subsequently continuously stored at − 80 °C until analysis.

### Blood serum and plasma analysis

Plasma glucose concentrations were analyzed with a colorimetric assay (GLUC3, Cobas, Roche Diagnostics GmbH, Mannheim, Germany) on an automated discrete analyzer (Cobas Mira, Roche Diagnostics GmbH, Mannheim, Germany). Serum insulin concentrations were analyzed by an equine-optimized ELISA (Equine Insulin ELISA, Mercodia, Uppsala, Sweden) previously validated for use in horses [7].

Plasma metabolome analysis was performed on all samples by the AbsoluteIDQ p180 Kit (Biocrates Life Science AG, Innsbruck, Austria) in the laboratory of Biocrates Life Science AG, according to the manufacturer’s standard protocol. This kit format-targeted metabolomics measurement was used to identify and quantify 188 metabolites belonging to five compound classes: acylcarnitines (40), proteinogenic and modified amino acids (19), glycerophospho- and sphingolipids (76 phosphatidylcholines, 14 lyso-phosphatidylcholines, 15 sphingomyelins), biogenic amines (19) and hexoses (1). A detailed list of the compounds is shown in the supplementary material (Additional file 1: Online Resource 1). The fully automated assay was based on phenylisothiocyanate derivatization in the presence of internal standards followed by FIA-MS/MS [acylcarnitines, (lyso-) phosphatidylcholines, sphingomyelins, hexoses] and LC-MS/MS (amino acids, biogenic amines) using a SCIEX 4000 QTRAP® (SCIEX, Darmstadt, Germany) or a Xevo TQ-S Micro (Waters, Vienna, Austria) instrument with electrospray ionization. The experimental metabolomics measurement technique is described in detail by patent US 2007/0004044 [8]. All pre-analytical and analytical procedures were performed, documented and reviewed according to the ISO 9001:2008 certified in-house quality management rules and guidelines of Biocrates Life Sciences AG.

### Data analysis and visualization

Plasma glucose and serum insulin concentrations of BASAL, OGT-120 and OGT-180 samples were used as a marker of insulin sensitivity status to assign horses to either of the two experimental groups: IS or ID. This was conducted by a hierarchical clustering algorithm using the Euclidean distance measure and Ward’s clustering method in MetaboAnalyst 3.0 [9].

Plasma metabolome data (absolute concentrations of compounds) were analyzed in MetaboAnalyst after normalization by Pareto scaling. All data were tested and confirmed to be normally distributed by use of the Shapiro-Wilk test in GraphPad Prism (GraphPad Prism, Version 6.07 for Windows, Graph-Pad Inc. La Jolla, CA). Principal component analysis (PCA), repeated measures two-way ANOVA (rmTWA) and heatmap generation were conducted in MetaboAnalyst to visualize and evaluate differences in the metabolic profiles due to classification according to insulin sensitivity status and during OGT. The heatmap was created for the top 22 significant metabolites identified by rmTWA; clustering of the metabolites by ‘average’ clustering algorithm and plotting the tree by Euclidean distance measure were also included. The RmTWA was performed for the factor ‘OGT’ (BASAL vs. OGT-120 vs. OGT-180) and ‘insulin sensitivity status’ (IS vs. ID), also considering interactions and applying false discovery rate correction. *P* ≤ 0.05 was considered as statistically significant. A volcano plot was created separately for the non-challenged condition (BASAL) to identify metabolites of interest, searching for metabolites having significantly different concentrations between IS and ID horses, even without any OGT influence. Metabolites with a fold change of at least 1.5 and a t-test *p* value lower than 0.05 were considered to have high importance. Concentrations of important metabolites were plotted in GraphPad Prism. These were tested statistically for the effect of ‘insulin sensitivity status’ at different levels of the OGT by rmTWA combined with a pairwise Bonferroni post hoc test in GraphPad Prism.

## Results

**Fig. 1:**
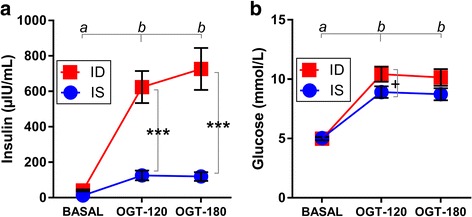
Increase of (**a**) serum insulin (repeated measure two-way ANOVA (rmTWA) factor oral glucose testing (OGT) *p* < 0.001) and of (**b**) glucose (rmTWA factor OGT *p* < 0.001) concentration in insulin-sensitive (IS) and insulin-dysregulated (ID) horses during OGT. Measurements were carried out at three levels of OGT: immediately before glucose administration (BASAL), and 120 min (OGT-120) and 180 min (OGT-180) after administration. Means ± SEM are shown, *n* = 10, significant differences between IS and ID are indicated as ***(*p* < 0.001), and a trend between IS and ID is indicated as ^**+**^(0.05 < *p* < 0.1). a, b = different superscripts indicate significant differences between basal and challenged conditions as the main OGT effect in both ID and IS horses (Bonferroni post hoc test)

All horses tolerated the OGT procedure without complications. Plasma glucose and serum insulin increased significantly as a response to oral glucose administration observed after 120 and 180 min of the OGT (Fig. 1a+b; both *p* < 0.001). The clustering algorithm applied to these data divided the horses clearly into two groups, which was the basis to assign them to either the IS (*n* = 10) or ID (*n* = 10) group. The IS group consisted of horses that had low to medium insulin concentrations in response to OGT, while the ID group consisted of horses that had high insulin concentrations in response to OGT. After creating the groups by clustering, the insulin threshold between the two groups was empirically found to be 260 μIU/mL with mean OGT-stimulated insulin concentrations of 131.5 ± 24.8 μIU/mL for IS (mean ± SEM; min. 34.5, max. 257.0 μIU/mL; *n* = 10) and 675.2 ± 101.2 μIU/mL for ID (mean ± SEM; min. 398.9, max. 1403.0 μIU/mL; *n* = 10), respectively (Mann-Whitney test *p* < 0.0001). The two groups had comparable BCS values as an indicator of obesity (6.8 ± 0.4 vs. 7.1 ± 0.2; mean ± SEM; *n* = 10/group), comparable BW (in kg: 481.1 ± 29.0 vs. 408.8 ± 53.72; mean ± SEM, *n* = 10/group) and similar ages (in years: 14.2 ± 1.6 vs. 15.7 ± 1.7; mean ± SEM; *n* = 10/group). The OGT-triggered increase in insulin concentration was significantly greater in ID horses compared to IS horses (Fig. 1a; *p* < 0.001), but the increase in glucose concentration was only slightly higher in ID horses (Fig. 1b; *p* = 0.98).

**Fig. 2:**
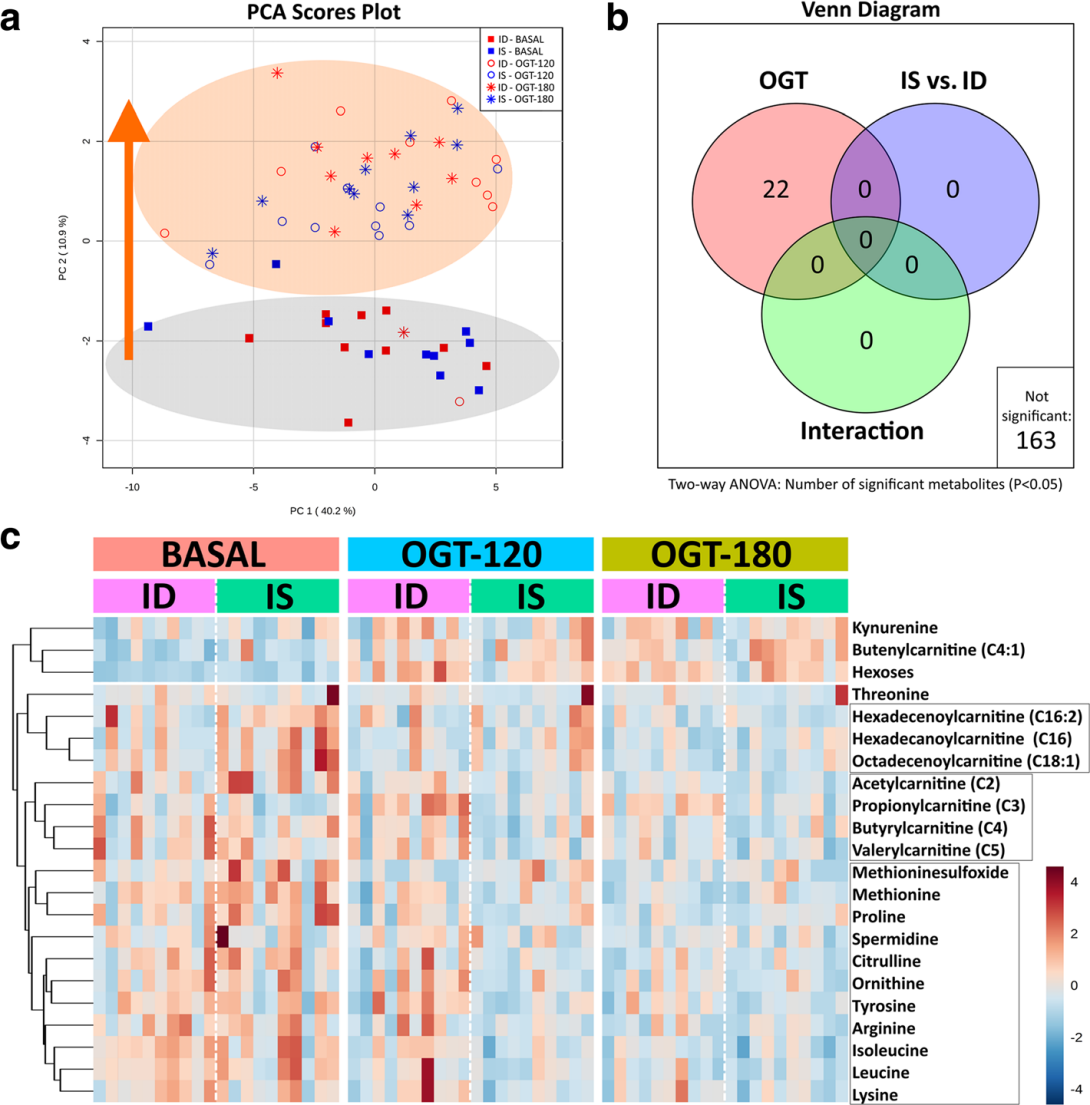
**a** Principal component analysis scores plot showing metabolic profiles of insulin-sensitive (IS) and insulin-dysregulated (ID) horses sampled before glucose administration (BASAL), and 120 min (OGT-120) and 180 min (OGT-180) after administration. **b** Number of significant metabolites detected by repeated measure two-way ANOVA with false discovery rate correction, analyzing the effect of OGT, insulin sensitivity status (IS vs. ID) and respective interactions. **c** Heatmap illustrating relative concentrations of top 22 significant metabolites during OGT: BASAL, OGT-120 and OGT-180 for IS and ID horses

**Fig. 3:**
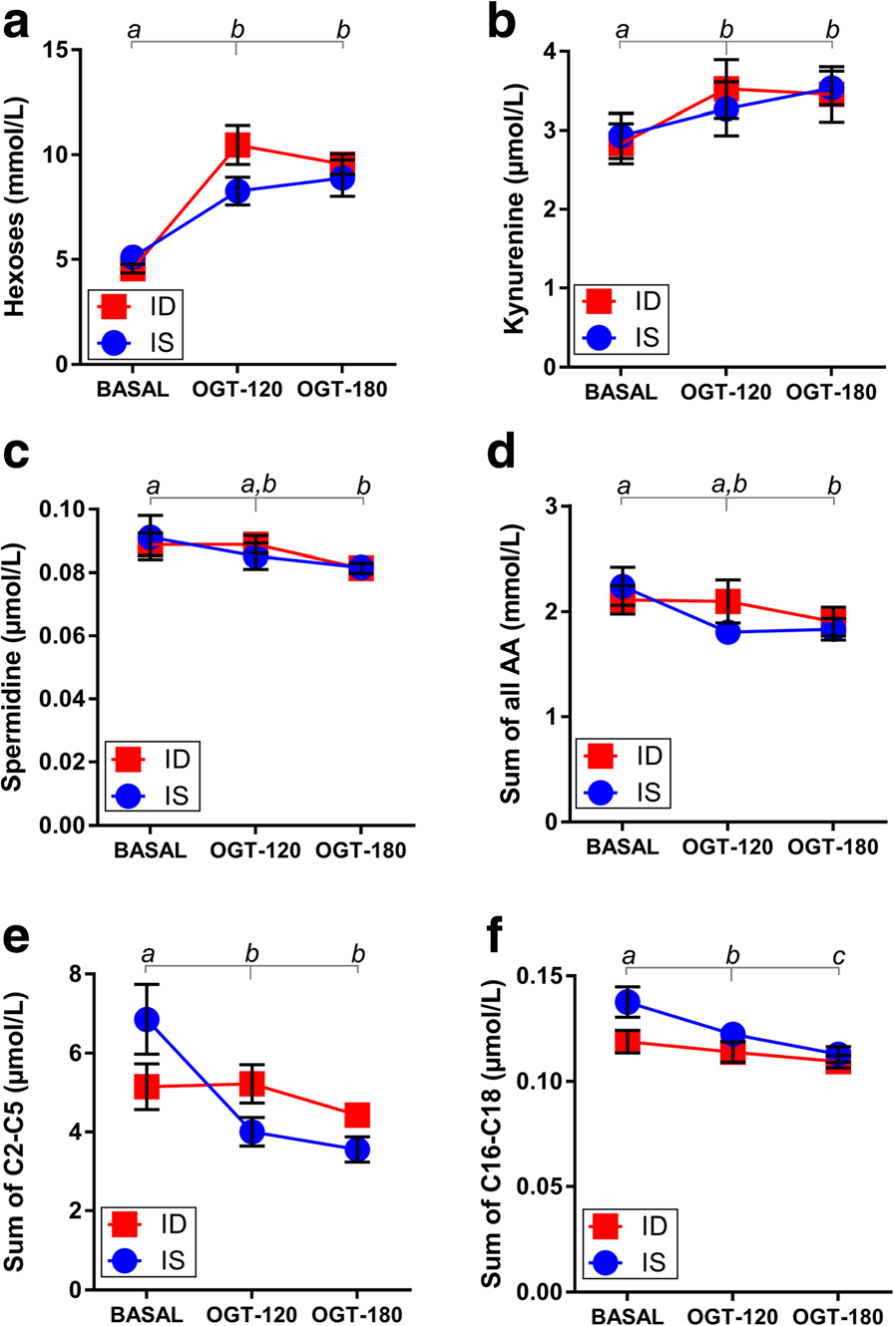
Metabolites of importance which were significantly affected by OGT in insulin-sensitive (IS) and insulin-dysregulated (ID) horses. Concentration of (**a**) hexoses (repeated measure two-way ANOVA (rmTWA), factor OGT *p* < 0.001) and (**b**) kynurenine (rmTWA, factor OGT *p* < 0.01) increased, while (**c**) spermidine (rmTWA, factor OGT *p* < 0.01), (**d**) sum of all proteinogenic amino acids (rmTWA, factor OGT *p* < 0.05) (**e**) sum of short-chain (C2, C3, C4 and C5) acylcarnitines (rmTWA, factor OGT *p* < 0.001) and (**f**) sum of long-chain (C16, C16:1, C16:2, C:18, C18:1, C:18:2) acylcarnitines (rmTWA, factor OGT *p* < 0.001) decreased. Insulin status had no significant effect. Means ± SEM are shown, *n* = 10; a, b, c = different superscripts indicate significant differences between basal and challenged conditions as the main OGT effect in both ID and IS horses (Bonferroni post hoc test)

An overview of the changes in the total metabolomic profiles of IS and ID horses during the OGT is given in Fig. 2. The PCA was used to downscale the dimensions of the data matrix resulting in PC1 and PC2 scores. The PCA scores plot showed a clear separation between the BASAL and the OGT-120/OGT-180 metabolic profiles with a shift of all OGT-treated horses along the y-axis (PC2; Fig. 2a). Twenty-two metabolites were significantly affected by the OGT (Fig. 2b), which was the underlying cause of the shift observed in the PCA. The insulin sensitivity status did not affect the metabolite profile, and interaction between the two factors also remained nonsignificant (Fig. 2b). Accordingly, the remaining 163 metabolites studied did not show any significant differences by ANOVA (Fig. 2b). A heatmap was created by using the 22 metabolites identified by the ANOVA to visualize the metabolites responsible for the OGT effect (Fig. 2c). The first level of branches in the clustering tree separated between 3 metabolites having increasing concentrations and 19 metabolites having decreasing concentrations during the OGT. The former group consisted of kynurenine, butenylcarnitine and hexoses, while the latter group consisted of long-chain fatty acylcarnitines (C16-18), short-chain fatty acylcarnitines (C2-5), (modified) amino acids and biogenic amines. Hexose (Fig. 3a; *p* < 0.001) and kynurenine (Fig. 3b; *p* = 0.002) concentrations were increased at OGT-120 and OGT-180, whereas spermidine (Fig. 3c; *p* = 0.006) concentrations decreased during OGT in both IS and ID horses. Proteinogenic amino acids (Fig. 3d; *p* = 0.024), short-chain acylcarnitines (Fig. 3e; *p* < 0.001) and long-chain acylcarnitines (Fig. 3f; *p* < 0.001) also decreased during OGT.

**Fig. 4:**
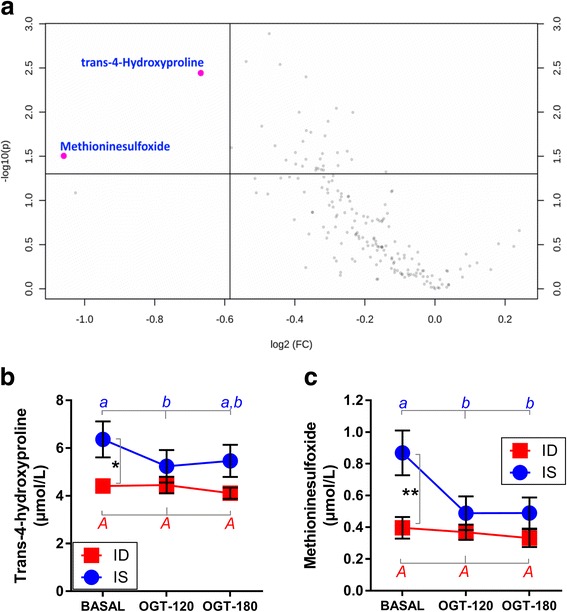
**a** Volcano plot highlighting metabolites of importance (trans-4-hydroxyproline *p* = 0.0036; methinonine sulfoxide *p* = 0.0134) which were different between insulin-sensitive (IS) and insulin-dysregulated (ID) horses under unchallenged conditions (only BASAL) (**b**) Trans-4-hydroxyproline (repeated measure two-way ANOVA (rmTWA), factor insulin sensitivity status *p* < 0.05, factor OGT *p* = 0.06) and (**c**) methionine sulfoxide (rmTWA, factor insulin sensitivity status *p* < 0.05, factor OGT *p* = 0.06) concentrations shown in serum of IS and ID horses at three levels of OGT: before glucose administration (BASAL), and 120 min (OGT-120) and 180 min (OGT-180) after administration (means ± SEM, *n* = 10). Significant differences between IS and ID are indicated as ** (*p* < 0.01), * (*p* < 0.05). a, b = different superscripts indicate significant differences (*p* < 0.05) between basal and challenged conditions in IS horses, while no OGT effect was observed (as indicated by A) in ID horses (Bonferroni post hoc test)

A volcano plot comprised of fold change and t-test statistics was analyzed using the results of BASAL samples to identify the metabolites with the greatest discriminating potential between IS and ID horses (Fig. 4a). This plot shows in its upper left corner the metabolites of interest (MOI), those that have the highest fold change and the lowest *p* value of the t-test comparing IS vs. ID. Two metabolites were clearly identified: trans-4-hydroxyproline (FC = 1.59, *p* = 0.003) and methionine sulfoxide (FC = 2.09, *p* = 0.013), with higher concentrations in IS horses compared to ID horses. Further assessment of the dynamic changes of these MOI during the OGT revealed that both trans-4-hydroxyproline (Fig. 4b) and methionine sulfoxide (Fig. 4c) concentrations decreased significantly in the IS group, but not in the ID group. Analyzing the effect of insulin sensitivity status by Bonferroni post hoc test revealed that these metabolites had significant differences only under BASAL conditions, with lower concentrations in the ID group (Fig. 4b: trans-4-hydroxyproline *p* = 0.040; Fig. 4c: methionine sulfoxide *p* = 0.010).

## Discussion

This study used a targeted and quantitative metabolomics analysis to describe novel metabolic profiles associated with equine insulin dysregulation in two approaches. (1) Basal concentrations before OGT challenge are suitable for identifying metabolites which can predict the metabolic characteristics of horses associated with insulin sensitivity or dysregulation. The dynamic responses after oral glucose load seen at OGT-120 and OGT-180 enables the identification of pathways which are causally affected by the oral glucose load as a single challenge in both IS and ID horses. The ID individuals have a higher pancreatic insulin secretion in response to a certain glucose load than IS individuals [1, 2, 6]. These dynamic changes of glucose and insulin were – as expected – clearly visible at 120 (OGT-120) and 180 (OGT-180) min after oral glucose load in the present study, confirming the classification into two groups (IS and ID) by the clustering algorithm. The threshold used here is considered independent of the cut-offs used clinically based on insulin concentrations during OGT and was declared as a differentiation criterion applicable to the animal cohort used in the current study to establish two experimental groups with a physiological and a pathophysiological response, respectively. Therefore, it should be emphasized that the metabolites identified here cannot be extrapolated for clinical diagnostic use; our findings provide rather a basis for hypotheses generation concerning insulin dysregulation mechanisms and potential candidates for future biomarker development. Moreover, the OGT induced a severe insulin increase with a wide dynamic range, reflecting that insulin sensitivity is not a dichotomous state of being either IS or ID, but rather that ID exists in different intensities. The typical condition of hyperinsulinemia was intensive in several ID horses, but this was not accompanied by a correspondingly strong hyperglycemia. This equine-specific pathophysiological phenomenon requires future clarification regarding its underlying mechanisms.

The corresponding metabolic profiles were studied by using the AbsoluteIDQ p180 Kit (Biocrates Life Science AG). This targeted metabolomics assay of kit format was designed by the developer to quantify a defined set of metabolites reflecting specific metabolic disorders, such as obesity, insulin resistance, proinflammation, dysregulation of glucose and lipid metabolism, and mitochondrial dysfunction. Although this assay was developed for human purposes, it is a species-independent analytical tool to accurately detect small molecules in various biological samples and, accordingly, we were able to detect a wide range of circulating acylcarnitines, amino acids, biogenic amines, glycerophospho- and sphingolipids, and hexoses in horses. The assay kit applied was considered suitable for acquiring phenotypic patterns of equine metabolism affected by insulin dysregulation because the metabolic syndrome of horses has been demonstrated to exhibit some common symptoms with the human metabolic syndrome: adiposity, chronic inflammation and mitochondrial dysfunction [1, 2].

### Metabolic response to oral glucose challenge

Metabolomic profiles changed overall after the OGT in a similar manner, irrespective of insulin sensitivity status and the time after challenge. Twenty-two metabolites were significantly affected by the OGT. The most obvious pattern seen was the rise of the hexose concentration at OGT-120 and OGT-180 in both IS and ID horses. Since circulating hexoses in adult horses consist mostly of glucose, this increase was expected after the OGT and was in accordance with the result of the serum glucose measurement.

Kynurenine concentrations were also increased due to the OGT in both IS and ID horses. Kynurenine is a known proinflammatory marker originating from tryptophan metabolism, which is increased in human patients with diabetic retinopathy [10]. Kynurenine is generated by indoleamine 2,3-deoxygenase, a rate-limiting enzyme, which is activated by inflammatory signals and reactive oxygen species (ROS) related to obesity and insulin resistance [10, 11]. In general, most of the ID horses are reported to be obese [1, 2]; however, the IS and ID horses in this study had comparable BCS values (as an indicator of obesity) and BW. Adipose tissue in humans was identified as a major source of indoleamine 2,3-deoxygenase activity and inflammatory cytokines [10]. Furthermore, acute short-term oral glucose load led to an inflammatory profile even in nondiabetic humans [12]. Although inflammatory cytokine levels were not determined in the horses of this study, the elevated kynurenine concentrations probably indicate a proinflammatory effect of short-term high glucose load by OGT. Therefore, the acute oral glucose load by OGT appeared to affect the molecular health of the horses and was also reflected by decreased spermidine concentrations in both IS and ID horses. Spermidine is a polycationic biogenic amine which is produced by various tissues. One of its predominant molecular functions is to inhibit nonenzymatic glycation of proteins and nucleic acids [13]. The strong reduction in spermidine concentrations indicated its utilization in the prevention of glycation-derived damage of molecules. To summarize, OGT with 1.0 g glucose/kg BW in horses, even as a short-term challenge, can potentially trigger a shift towards a proinflammatory metabolic phenotype.

Administering glucose in high amounts by OGT necessarily induced a shift in substrate availability for energy-gaining pathways. Horses are known to have a well-developed short-chain fatty acid (SCFA) metabolism, with large amounts of butyrate absorbed from the colon [14]. Thus, their energy metabolism depends largely on SCFA, while glucose must be produced by gluconeogenesis from gluconeogenic amino acids, at least when fed on a diet rich in crude fiber and low in grain. During OGT, gluconeogenic needs decreased and the excess of glucose could be used for ATP generation, while utilization of long-chain fatty acids was diminished, a physiological effect called the Randle cycle [15]. Increasing insulin levels due to the OGT probably accounted for short-term protein anabolism. This was reflected in the metabolomics approach in IS and ID horses by a decrease of all proteinogenic amino acids. A reduced utilization of SCFA was reflected by a decrease of short-chain acylcarnitines, mitochondrial degradation products derived mainly from SCFA metabolism. Moreover, enhanced insulin-mediated anti-lipolysis was reflected by reduced concentrations of long-chain acylcarnitines, mitochondrial degradation products of long-chain fatty acids.

### Novel metabolites and pathways associated with insulin resistance

No clear differences between IS and ID horses (if present) could be detected by ANOVA, PCA or the heatmap because of the dominance of the OGT effect on the total metabolome. Physiologically, this means that the response to glucose was equally intensive in both IS and ID horses, superposing any potential effects of insulin dysregulation. However, the metabolomics approach was also performed to identify the basal differences of metabolic profiles as a function of insulin sensitivity status.

Therefore, a volcano plot comprising fold change and t-test statistics was analyzed using the results of BASAL samples to identify the metabolites with the greatest discriminating potential between IS and ID horses. Trans-4-hydroxyproline and methionine sulfoxide attracted attention, with higher concentrations in IS horses compared to ID horses. During the OGT, both MOI, trans-4-hydroxyproline and methionine sulfoxide, decreased significantly in the IS group, but not in the ID group. However, the concentration of these metabolites was only different between IS and ID under BASAL conditions, with lower concentrations in the ID group. It is suggested that both MOI belong to the oxidant–antioxidant system of an organism to cope with oxidative stress directly (methionine sulfoxide) or indirectly (trans-4-hydroxyproline; synthesis needs vitamin C, an antioxidant) [11, 16, 17]. Oxidative stress is defined as oxidant–antioxidant disequilibrium due to either increased ROS production, a decrease in the capacity of antioxidant system to defend the organisms against ROS or both. The ROS promote cell damage and can modify endocrine signaling pathways, such as the insulin response cascade leading to insulin resistance [18], one of the potential underlying processes for insulin dysregulation. Disturbances of the oxidant–antioxidant equilibrium have been well-studied in horses suffering from various diseases, but decent markers are still not sufficiently characterized [19, 20]. It is especially not clear yet whether a disequilibrium of the oxidant–antioxidant system is causally relevant for the development of insulin dysregulation in horses. Equine insulin dysregulation and disturbances in the oxidant–antioxidant system are currently understood poorly. Banse et al. [21] suggested that obesity-associated hyperinsulinemia was connected with oxidative stress in skeletal muscle, but they did not find any evidence for oxidative damage to skeletal muscle in obese hyperinsulinemic horses. However, protein carbonyls – products of protein oxidation by ROS – were significantly reduced in the skeletal muscle of obese hyperinsulinemic horses [21]. Consistent with these findings, both MOI found in this study decreased with increasing glucose and insulin concentrations due to the OGT in serum, with a more pronounced decrease in IS horses. Accepting the former suggestion that acute oral glucose load causally promoted a shift into a more inflammatory status even in healthy horses, a decrease in plasma markers of oxidative stress might reflect an increasing disequilibrium in tissue oxidant–antioxidant balance in IS horses; this condition is intensified in ID horses.

## Conclusion

It can be concluded as a working hypothesis that higher concentrations of trans-4-hydroxyproline and methionine sulfoxide under basal conditions are a positive sign for health and insulin sensitivity, indicating that healthy horses can remove oxidatively modified amino acids from cells more effectively to avoid cell damage. The lower basal values of these MOI and their decrease during OGT, as well as the concurrent increase of the proinflammatory marker kynurenine are assessed as negative signs and indicate that oxidative stress is involved in the pathway to insulin dysregulation of horses. However, the underlying mechanisms by which oxidized amino acids were produced and modulated in equine tissues is widely unknown so far. Furthermore, because of the small sample size (*n* = 10 per group) and the lack of a clinical diagnosis, the potential significance of trans-4-hydroxyproline and methionine sulfoxide as biomarkers for equine insulin dysregulation has to be tested in larger populations under clinical settings. However, the high diversity of the experimental horse population presented regarding breed, age and BW suggests a universal pathophysiological potential for equine insulin dysregulation. Further research based on these implications should focus on defining criteria for the early diagnosis of insulin dysregulation in horses.

## Additional file

Additional file 1: Online Resource 1. List of metabolites measured by the Absolute-IDQ p180 Kit. Name and abbreviation of metabolites measured in the targeted metabolomics analysis approach by using the Absolute-IDQ p180 Kit of Biocrates Life Sciences AG (Innsbruck, Austria). (DOCX 17 kb)

## Abbreviations

ANOVA: Analysis of variance
BCS: Body condition score
BW: Body weight
FIA-MS/MS: Flow-injection analysis and tandem mass spectrometry
ID: Insulin-dysregulated
IS: Insulin-sensitive
LC-MS/MS: Liquid chromatography and tandem mass spectrometry
MOI: Metabolite of interest
OGT: Oral glucose test
rmTWA: Repeated measures two way analysis of variance
ROS: Reactive oxygen species
SCFA: Short-chain fatty acids
SEM: Standard error of the mean
