# Supplementary material for: Lower plasma trans-4-hydroxyproline and methionine sulfoxide levels are associated with insulin dysregulation in horses
Source: BMC Vet Res. 2018 May 2;14:146. doi: 10.1186/s12917-018-1479-z (PMC5930486; doi:10.1186/s12917-018-1479-z)
Supplement: Supplementary file 1 — Online Resource 1. List of metabolites measured by the Absolute-IDQ p180 Kit. Name and abbreviation of metabolites measured in the targeted metabolomics analysis approach by using the Absolute-IDQ p180 Kit of Biocrates Life Sciences AG (Innsbruck, Austria). (DOCX 17 kb) [file 12917_2018_1479_MOESM1_ESM.docx]

**Online Resource 1.** List of metabolites measured by the Absolute-IDQ p180 Kit

| **Compound class** | | **Metabolites** |
| --- | --- | --- |
| (A) | Acylcarnitines | Carnitine (C0), Acetylcarnitine (C2), Propionylcarnitine (C3), Propenoylcarnitine (C3:1), Hydroxypropionylcarnitine (C3-OH), Butyrylcarnitine (C4), Butenylcarnitine (C4:1), Hydroxybutyrylcarnitine (C4-OH (C3-DC)), Valerylcarnitine (C5), Tiglylcarnitine (C5:1), Glutaconylcarnitine (C5:1-DC), Glutarylcarnitine (Hydroxyhexanoylcarnitine) (C5-DC (C6-OH)), Methylglutarylcarnitine (C5-M-DC), Hydroxyvalerylcarnitine (Methylmalonylcarnitine) (C5-OH (C3-DC-M)), Hexanoylcarnitine (Fumarylcarnitine) (C6 (C4:1-DC)), Hexenoylcarnitine (C6:1), Pimelylcarnitine (C7-DC), Octanoylcarnitine (C8), Nonaylcarnitine (C9), Decanoylcarnitine (C10), Decenoylcarnitine (C10:1), Decadienylcarnitine (C10:2), Dodecanoylcarnitine (C12), Dodecenoylcarnitine (C12:1), Dodecanedioylcarnitine (C12-DC), Tetradecanoylcarnitine (C14), Tetradecenoylcarnitine (C14:1), Hydroxytetradecenoylcarnitine (C14:1-OH), Tetradecadienylcarnitine (C14:2), Hydroxytetradecadienylcarnitine (C14:2-OH), Hexadecanoylcarnitine (C16), Hexadecenoylcarnitine (C16:1), Hydroxyhexadecenoylcarnitine (C16:1-OH), Hexadecadienylcarnitine (C16:2), Hydroxyhexadecadienylcarnitine (C16:2-OH), Hydroxyhexadecanoylcarnitine (C16-OH), Octadecanoylcarnitine (C18), Octadecenoylcarnitine (C18:1), Hydroxyoctadecenoylcarnitine (C18:1-OH), Octadecadienylcarnitine (C18:2) |
| (B) | Amino acids | Alanine (Ala), Arginine (Arg), Asparagine (Asn), Aspartate (Asp), Citrulline (Cit), Glutamine (Gln), Glutamate (Glu), Glycine (Gly), Histidine (His), Isoleucine (Ile), Leucine (Leu), Lysine (Lys), Methionine (Met), Ornithine (Orn), Phenylalanine (Phe), Proline (Pro), Serine (Ser), Threonine (Thr), Tryptophan (Trp), Tyrosine (Tyr), Valine (Val) |
| (C) | Biogenic amines | Acetylornithine (Ac-Orn), Asymmetric dimethylarginine (ADMA), Symmetric dimethylarginine (SDMA), alpha-Aminoadipic acid (alpha-AAA), Carnosine (Carnosine), Creatinine (Creatinine), Histamine (Histamine), Kynurenine (Kynurenine), Methioninesulfoxide (Met-SO), Nitrotyrosine (Nitro-Tyr), cis-4-Hydroxyproline (cis-OH-Pro), trans-4-Hydroxyproline (trans-OH-Pro), Phenylethylamine (PEA), Putrescine (Putrescine), Sarcosine (Sarcosine), Serotonin (Serotonin), Spermidine (Spermidine), Spermine (Spermine), Taurine (Taurine), Dopamine (Dopamine), DOPA (DOPA) |
| (D) | Glycerophospholipids |  |
|  | Lyso-PC | lysoPC a C14:0, lysoPC a C16:0, lysoPC a C16:1, lysoPC a C17:0, lysoPC a C18:0, lysoPC a C18:1, lysoPC a C18:2, lysoPC a C20:3, lysoPC a C20:4, lysoPC a C24:0, lysoPC a C26:0, lysoPC a C26:1, lysoPC a C28:0, lysoPC a C28:1 |
|  | Diacyl-PC | PC aa C24:0, PC aa C26:0, PC aa C28:1, PC aa C30:0, PC aa C30:2, PC aa C32:0, PC aa C32:1, PC aa C32:2, PC aa C32:3, PC aa C34:1, PC aa C34:2, PC aa C34:3, PC aa C34:4, PC aa C36:0, PC aa C36:1, PC aa C36:2, PC aa C36:3, PC aa C36:4, PC aa C36:5, PC aa C36:6, PC aa C38:0, PC aa C38:1, PC aa C38:3, PC aa C38:4, PC aa C38:5, PC aa C38:6, PC aa C40:1, PC aa C40:2, PC aa C40:3, PC aa C40:4, PC aa C40:5, PC aa C40:6, PC aa C42:0, PC aa C42:1, PC aa C42:2, PC aa C42:4, PC aa C42:5, PC aa C42:6 |
|  | Acyl-alkyl-PC | PC ae C30:0, PC ae C30:1, PC ae C30:2, PC ae C32:1, PC ae C32:2, PC ae C34:0, PC ae C34:1, PC ae C34:2, PC ae C34:3, PC ae C36:0, PC ae C36:1, PC ae C36:2, PC ae C36:3, PC ae C36:4, PC ae C36:5, PC ae C38:0, PC ae C38:1, PC ae C38:2, PC ae C38:3, PC ae C38:4, PC ae C38:5, PC ae C38:6, PC ae C40:1, PC ae C40:2, PC ae C40:3, PC ae C40:4, PC ae C40:5, PC ae C40:6, PC ae C42:0, PC ae C42:1, PC ae C42:2, PC ae C42:3, PC ae C42:4, PC ae C42:5, PC ae C44:3, PC ae C44:4, PC ae C44:5, PC ae C44:6 |
| (E) | Sphingolipids | SM (OH) C14:1, SM (OH) C16:1, SM (OH) C22:1, SM (OH) C22:2, SM (OH) C24:1, SM C16:0, SM C16:1, SM C18:0, SM C18:1, SM C20:2, SM C22:3, SM C24:0, SM C24:1, SM C26:0, SM C26:1 |
| (F) | Hexoses | Sum of hexoses (H1) |

PC: phosphatidylcholines; SM: sphingomyelins
